# The S2 Subunit of QX-type Infectious Bronchitis Coronavirus Spike Protein Is an Essential Determinant of Neurotropism

**DOI:** 10.3390/v11100972

**Published:** 2019-10-22

**Authors:** Jinlong Cheng, Ye Zhao, Gang Xu, Keran Zhang, Wenfeng Jia, Yali Sun, Jing Zhao, Jia Xue, Yanxin Hu, Guozhong Zhang

**Affiliations:** Key Laboratory of Animal Epidemiology of the Ministry of Agriculture, College of Veterinary Medicine, China Agricultural University, Beijing 100193, Chinahvbhvgbhub@163.com (Y.Z.); xugangayu@163.com (G.X.); xuejia@cau.edu.cn (J.X.);

**Keywords:** coronavirus, infectious bronchitis virus, QX type, furin-S2′ site, encephalitis, neurotropism

## Abstract

Some coronaviruses (CoVs) have an extra furin cleavage site (RRKR/S, furin-S2′ site) upstream of the fusion peptide in the spike protein, which plays roles in virion adsorption and fusion. Mutation of the S2′ site of QX genotype (QX-type) infectious bronchitis virus (IBV) spike protein (S) in a recombinant virus background results in higher pathogenicity, pronounced neural symptoms and neurotropism when compared with conditions in wild-type IBV (WT-IBV) infected chickens. In this study, we present evidence suggesting that recombinant IBV with a mutant S2′ site (furin-S2′ site) leads to higher mortality. Infection with mutant IBV induces severe encephalitis and breaks the blood–brain barrier. The results of a neutralization test and immunoprotection experiment show that an original serum and vaccine can still provide effective protection in vivo and in vitro. This is the first demonstration of IBV-induced neural symptoms in chickens with encephalitis and the furin-S2′ site as a determinant of neurotropism.

## 1. Introduction

Coronaviruses (CoVs) are single-strand plus RNA viruses, which belong to the order *Nidovirales*. CoVs normally infect epithelial cells such as respiratory epithelial cells, intestinal epithelial cells and kidney epithelial cells, which causes respiratory or intestinal tract diseases with various levels of severity [1,2]. Human coronavirus 229E (HCoV-229E) is a main cause of the common cold, while severe acute respiratory syndrome coronavirus (SARS-CoV) and Middle East respiratory syndrome coronavirus (MERS-CoV) cause severe respiratory disease that can be life-threatening to humans [3,4,5]. However, some CoVs-like mouse hepatitis virus John Howard Mueller strain (MHV-JHM) infect oligodendrocytes, astrocytes and microglia and cause acute encephalomyelitis [6,7]. Additionally, human coronavirus OC43 (HCoV-OC43) can also reach the central nervous system (CNS) by intranasal inoculation in BALB/c mice, which causes encephalitis [8,9,10,11]. The release of cytokines such as TNF-α, interleukin-1β (IL-1β) and interleukin-6 (IL-6) is closely associated with encephalitis [12]. Infectious bronchitis virus (IBV) belongs to the gamma-coronavirus group (γ-CoV), which causes a highly contagious, acute viral respiratory disease of chickens that has caused great economic losses in the poultry industry. At present, QX-type IBV is epidemic worldwide; it can damage the respiratory, digestive and urogenital systems of chickens [13,14,15,16]. Besides, because of severe damage of respiratory mucosa, infected chickens are susceptible to secondary infection by mycoplasma, bacteria, or other pathogens causing high mortality.

All CoV virions encode a set of four structural proteins, namely; spike glycoprotein (S), small envelope protein (E), membrane glycoprotein (M) and nucleocapsid proteins (N) [17,18]. S protein belongs to the class I fusion proteins, located at the surface of the virion [19]. The trimeric S glycoprotein is a class I fusion protein and mediates attachment to the host receptor. S is typically cleaved by a host cell furin-like protease into two separate polypeptides named S1 and S2. S1 makes up the large receptor-binding domain of the S protein, while S2 forms the stalk of the spike molecule [20]. There is an extra furin cleavage site (furin-S2′ site) upstream of the fusion peptide in the S2 subunit of some CoVs, which has been reported to influence the mechanism of invasion of host cells by CoVs. When the original sequence PTKR/S of SARS-CoV was replaced with RRKR/S, SARS-CoV could invade susceptible cells while no longer relying on cathepsin L [21]. Additionally, after the S2′ site mutated into the furin-S2′ site of the MHV-A59 strain, MHV can fuse with susceptible cells in the early endosome, but not lysosome, without requiring lysosomal enzymes such as MERS-CoV [22]. As for IBV, the furin-S2′ site is a determinant of cellular adaptation of the IBV-Beaudette strain. If this motif is lacking, the IBV-Beaudette strain, which is normally the only IBV strain able to adapt to the Vero cell line, can no longer grow in these cells [23,24,25].

Most basic research on IBV performed to date has been based on the IBV-Beaudette strain; therefore, much remains unknown about the pathogenic mechanism of QX-type IBV. Here, we rescued a mutant QX-type IBV with the furin-S2′ site and compared the pathogenicity of the mutant IBV with its parental virus. We also further investigated the tissue and cell tropism. Finally, we clarified the biological significance with an immunoprotection experiment. The function of S protein in QX-type IBV was demonstrated, which is more valuable for poultry than research about the IBV-Beaudette strain.

## 2. Materials and Methods

### 2.1. Cells and Virus

CV-1 cells and D980R cells were used to assemble the full-length genome of mutant IBV in the vaccinia virus, cultured in Minimum Essential Medium (MEM; Thermo Fisher, Waltham, MA, USA) with 10% fetal bovine serum (FBS; Gibco, Grand Island, NY, USA). Baby hamster kidney (BHK) cells were used to electroporate to rescue the YN strain with furin-S2’ site (rYN-S2/RRKR). Chicken embryo kidney cells were prepared from 16-day-old SPF chicken embryos to measure the 50% tissue culture infectious dose (TCID_50_) of rYN and rYN-S2/RRKR. Chicken embryo kidney (CEK) cells were cultured in Dulbecco’s Modified Eagle Medium (DMEM, Thermo Fisher), supplemented with 10% FBS. IBV-rYN is an infectious molecular clone of IBV-YN strain (GenBank accession number: JF893452), which grows in CEK cells [26]. rYN-S2/RRKR is an infectious molecular clone of the IBV-YN strain whose sequence PRGR/S upstream of the FP was replaced with RRKR/S (furin-S2′ site). SZ130 is a QX-type attenuated IBV strain and an IBV vaccine candidate [27]. 

### 2.2. Generation of Recombinant Virus

Recombinant rYN-S2/RRKR virus containing an S protein with the furin-S2′ site was generated by vaccinia recombination, as described previously [20,28]. Briefly, plasmid with the furin-S2′ site was generated using the Seamless Assembly kit (Invitrogen, Carlsbad, CA, USA) and transfected into CV-1 cells infected by vaccinia virus containing the genome of YN-ΔS-GPT. Furin-S2’ site was introduced into the YN cDNA by homologous recombination using the transient dominant selection system [25]. After screening and transcription in vitro, the full-length RNA of rYN-S2/RRKR was electroporated into BHK cells. After 48 h of incubation at 37 °C, cells and supernatant were repeatedly subjected to freezing and thawing three times and inoculated into 10-day-old SPF embryonated eggs. Serial passage was executed until the stable appearance of dwarf embryos and the time of death of embryos was recorded. The 50% embryo infectious dose (EID_50_) was determined by inoculating serial 10-fold dilutions of allantoic fluid into SPF chicken embryos [29]. The TCID_50_ was determined by inoculating serial 10-fold dilutions of allantoic fluid into CEK cells cultured in DMEM with 10% FBS in 24-well plates.

### 2.3. Growth Curves of Parental and Recombinant Viruses

CEK cells were infected with 10^3.0^ EID_50_ of rYN or rYN-S2/RRKR in DMEM containing 10% FBS. After 1 h of infection, the supernatant was replaced with fresh DMEM with 10% FBS. Samples of cells and culture supernatant were collected at 1, 6, 12, 24, 36, 48 and 60 h post-inoculation (hpi). RNA was extracted from the samples using Cell Total RNA Isolation Kit (FORE GENE, Chengdu, China). The samples were subjected to RT-qPCR analysis to quantify virion production. The viral loads were determined with the Light Cycler 96 Real-Time PCR system. Specific primers against the IBV N gene was designed and we inserted the fragment into the pEASY-Blunt vector to generate a standard plasmid as previously described [13,30]. We generated a standard curve by plotting the cycle threshold (CT) values against the copy numbers of the standard plasmid. 

### 2.4. Animals and Ethics Statement

All SPF chickens and SPF embryonated eggs (10 days old) were purchased from Beijing Boehringer Ingelheim Vital Biotechnology Co. Ltd. (Beijing, China). All chickens were raised in isolators at China Agricultural University throughout the experiments with feed and water provided ad libitum. The treatment of all laboratory animals was approved by the Beijing Administration Committee of Laboratory Animals under the leadership of the Beijing Association for Science and Technology (approval ID SYXK [Jing] 2018-0038, 11/27/2018). The protocols of this experiment were performed in accordance with the guidelines of the Animal Welfare and Ethical Censor Committee at China Agricultural University (CAU approval number 19036, 12/10/2018). 

### 2.5. Pathogenicity Experiments

Sixty 1-day-old SPF chickens were randomly divided into three groups of 20 chickens each and kept in three isolators. Two of the three groups were inoculated with 100 μL of rYN or rYN-S2/RRKR strain containing 10^5.0^EID_50_ via the intraocular route. The third group was inoculated with 100 μL of phosphate-buffered saline (PBS) as a negative control. Ten chickens of each group were used only for observation, while the other ten were used for sampling. 

Thirty 3-week-old SPF chickens were divided randomly into three groups of 10 chickens and kept in three isolators. Two of three groups were inoculated with 100 μL of rYN or rYN-S2/RRKR strain containing 10^5.0^EID_50_ via the intraocular route. The third group was inoculated with 100 μL of PBS as a negative control. 

#### 2.5.1. Clinical Observations and Sampling

To determine the pathology of the parental and mutant IBV strains, all chickens were observed daily for 14 days and their clinical signs were recorded. Two chickens from each group were sacrificed at 3, 5, 7, 9 and 11 days post-inoculation (dpi). Gross lesions were noted, and samples of trachea, lung, kidney and brain were collected and stored in 10% neutral formalin for histopathological examinations.

#### 2.5.2. Histopathology and Immunohistochemistry (IHC)

The tissues collected as described above were immersed in 10% neutral formalin for 48 h. Fixed samples were processed, embedded in paraffin wax and cut into 5-μm sections. Sections were stained with hematoxylin and eosin and were examined by light microscopy for lesions. In the same tissue sections prepared for IHC, the intensity of viral antigen was detected. Five-micrometer sections were subjected to antigen retrieval and blocked by 10% normal goat serum in PBS for 30 min to eliminate nonspecific binding. Then, tissue sections were incubated with chicken anti-IBV hyperimmune serum at 1:500 dilution in PBS for 12 h at 4 °C; then, incubation with horseradish peroxidase-conjugated rabbit anti-chicken IgG and horseradish peroxidase-conjugated rabbit anti-mouse IgG (Sigma, St. Louis, MO, USA) was performed for 1 h. The reaction was visualized using 3,3-diaminobenzidine (DAB; Sigma) for 10 min. Finally, sections were counterstained with hematoxylin and examined by light microscopy. Sections stained with hematoxylin-eosin (H&E) were evaluated and the scores from 0 to 10, reflecting the severity of the lesions, were recorded. The indications for the scores were as follows: 0 = no microscopic lesions, 1–3 = mild lesions, 4–6 = moderate lesions and 7–10 = severe and extensive lesions. The detection of the IBV antigen was evaluated based on the number of positive cells per section from each microscopic field and is presented with a ranked score of 0–4. The scores represent the following: 0 = no positive cells, 1 = 1–10 positive cells, 2 = 11–30 positive cells, 3 = 31–50 positive cells and 4 = > 50 positive cells. 

### 2.6. Neutralization Test and Immunoprotection Experiment

To determine the influence on antigenicity of the furin-S2′ site, neutralization tests in vitro were performed as described previously [14]. Briefly, chicken anti-IBV-YN hyperimmune serum was inactivated at 56 °C for 30 min and then serially diluted two-fold. The diluted serum was incubated with the same volume of rYN or rYN-S2/RRKR containing 200 EID_50_ at 37 °C for 1 h. The virus–serum mixtures were then inoculated into the allantoic cavity of 10-day-old SPF chicken embryos. After 6 days, the embryos were examined for dwarfing. The 50% protective dose (PD_50_) was calculated by the Reed and Muench method. The virus–PBS mixtures were inoculated as mock control groups.

Fifty 1-day-old SPF chickens were divided into three groups, vaccinated with SZ130 containing 10^5.0^EID_50_ via the intraocular route. Two weeks later, two groups were inoculated with 100 μL of rYN or rYN-S2/RRKR 10^5.0^EID_50_. All chickens were observed for 2 weeks after infection.

## 3. Results

### 3.1. Decreased Replication of rYN-S2/RRKR in CEK Cells

**Figure 1:**
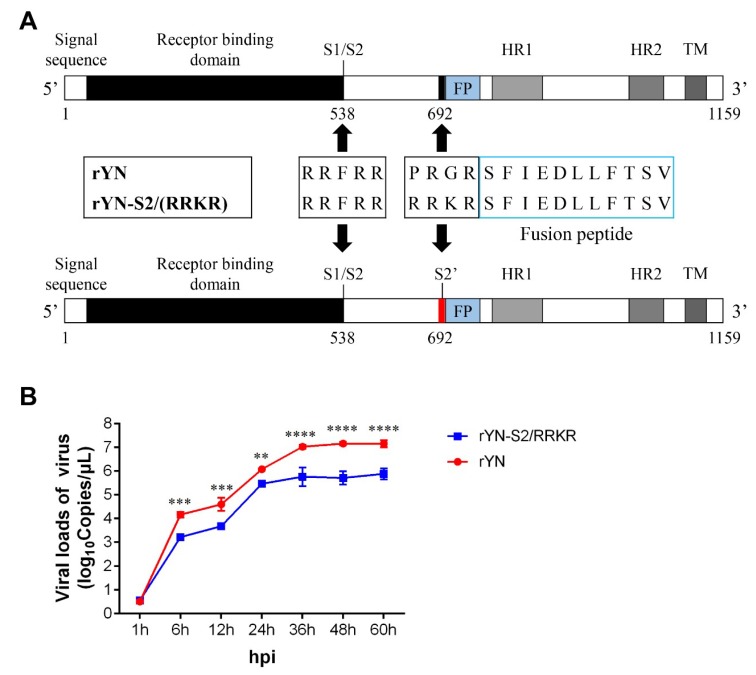
Introduction of a furin cleavage site upstream of the fusion peptide. (**A**) Schematic representation of the IBV spike protein. The original cleavage site at the S1/S2 boundary and a furin cleavage site upstream of the FP are indicated by an arrow. (**B**) Growth curves of rYN and rYN-S2/RRKR in CEK cells. Virus (10^3.0^EID_50_) was inoculated in the CEK cells for 60 h. Viral loads were determined by measuring expression of the N gene in cell lysates using absolute RT-qPCR (*n* = 3 × 3). Two-way ANOVA was used for analysis of differences in GraphPad Prism 7.0 and the significance was considered as follows: highly significant at *p* < 0.01 (**), very highly significant at *p* < 0.001 (***) and extremely significant at *p* < 0.0001 (****).

The S2′ site upstream of FP was mutated into the furin-S2′ site and recombinant virus was rescued successfully named rYN-S2/RRKR (Figure 1A). We found that rYN-S2/RRKR was highly fatal to 10-day-old SPF embryonated eggs compared with its parental rYN strain. rYN-S2/RRKR inoculation caused all embryonated eggs to die within 36 h post-inoculation (hpi), whereas this required more than 96 hpi in rYN (Table 1). Additionally, the 50% embryo infectious dose (EID_50_) of rYN-S2/RRKR in SPF embryonated eggs was approximately one-tenth that of rYN (10^6.5^EID_50_/0.1 mL for rYN-S2/RRKR versus 10^7.17^EID_50_/0.1 mL for rYN), while the 50% tissue culture infectious dose (TCID_50_) of rYN-S2/RRKR was more than 1000-fold higher than that of rYN (10^5.5^TCID_50_/0.1 mL for rYN-S2/RRKR versus 10^3.23^TCID_50_/0.1 mL for rYN). The decreased replication of rYN-S2/RRKR compared with that of its parental strain was further confirmed by analyzing the growth curve in CEK cells (Figure 1B).

### 3.2. rYN-S2/RRKR Causes Pronounced Encephalitis

#### 3.2.1. Clinical Signs and Gross Lesions in 1-Day-Old SPF Chicks

**Figure 2:**
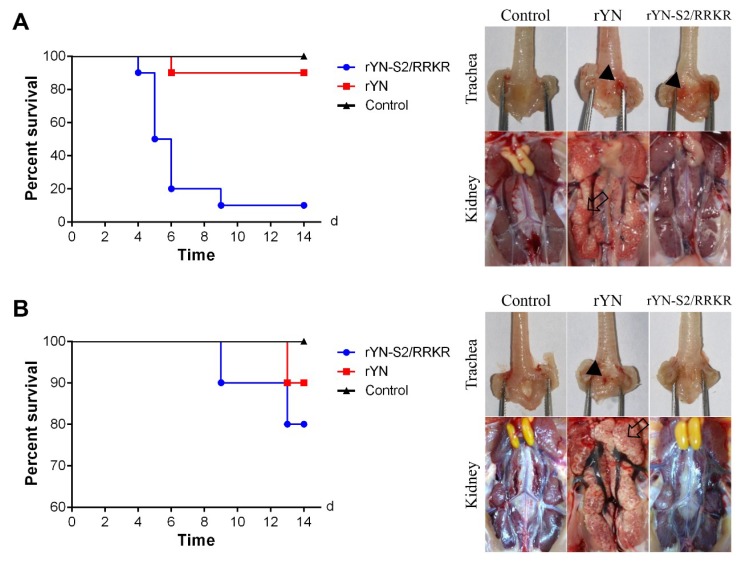
Furin-S2′ site can cause high mortality in SPF chicken without obvious gross kidney injury. (**A**) Survival rate of 1-day-old SPF chickens inoculated with rYN or rYN-S2/RRKR. rYN-S2/RRKR was highly pathogenic to 1-day-old SPF chickens, with a mortality rate of 90% (9/10), while the mortality rate of rYN was 10% during the observation period (left). Results of necropsy at 9 dpi on 1-day-old chickens inoculated with rYN or rYN-S2/RRKR. Mucus and punctate hemorrhage in the larynx and trachea (black triangles), substantial urate deposits in the ureter and kidney swelling were observed (blank arrow), while only mucus and punctate hemorrhage in the larynx were observed in the rYN-S2/RRKR group (right). (**B**) Survival rate of 3-week-old SPF chickens inoculated with rYN or rYN-S2/RRKR. Diseased chickens in the rYN-S2/RRKR group appeared later than in 1-day-old chickens and the mortality rate was 20% (2/10). The mortality of rYN was still 10%, but disease onset was delayed (left). At necropsy of diseased chickens at 13 dpi, mucus and punctate hemorrhage in the larynx (black triangles) and mottled swelling kidney (blank arrow) were observed in the rYN-inoculated group. There were no obvious gross lesions in the rYN-S2/RRKR group and negative control group (right).

Because the furin-S2′ site induction resulted in increased mortality associated with rYN in SPF embryonated eggs compared with that of its parental strain, we next evaluated the pathogenicity of rYN-S2/RRKR in 1-day-old SPF chickens. One-day-old SPF chicks inoculated with the rYN strain began to show clinical signs of sneezing and listlessness at 5 days post-inoculation (dpi). One chick died in the rYN-inoculated observation group during the experiment’s observation period, conferring a mortality rate of 10%, while chicks in the rYN-S2/RRKR-inoculated group began to show clinical signs of diarrhea and unexpectedly neurological signs such as head tremor and paralysis. Nine out of 10 chicks infected with rYN-S2/RRKR during the observation period; the mortality was 90% (Figure 2A, left panel). The survival results showed that the furin-S2′ site clearly increased the lethality of the rYN strain to 1-day-old SPF chickens. Interestingly, furin-S2′ site induction caused neurological signs that have never previously been reported. At necropsy of 1-day-old SPF chicks at 5, 7 and 9 dpi, there were obvious lesions in the respiratory and urinary systems of chicks inoculated with the rYN strain, including mucus and punctate hemorrhage in the larynx, substantial urate deposits in the ureter and kidney swelling. Chicks inoculated with rYN-S2/RRKR strain had mucus and punctate hemorrhage in the larynx and no obvious lesions in the kidney (Figure 2A, right panels). Additionally, no gross lesions were observed in the negative control group. Our results demonstrate that rYN-S2/RRKR was highly fatal to 1-day-old SPF chicks compared with its parental strain.

#### 3.2.2. Clinical Signs and Gross Lesions in 3-Week-Old SPF Chicks

To further evaluate the age dependence of pathogenicity caused by rYN-S2/RRKR, we performed pathogenicity experiments on 3-week-old SPF chicks. Chicks inoculated with the rYN strain began to exhibit listlessness at 11 dpi and one chick died at 13 dpi. The mortality rate of rYN was 10%. Conversely, chicks inoculated with rYN-S2/RRKR began to show neurological signs of head tremor but no paralysis at 8 dpi. The mortality rate of rYN-S2/RRKR in 3-week-old chicks was 20% (Figure 2B, left panel). At necropsy of dead 3-week-old chicks, chicks inoculated with the rYN strain showed the same findings as 1-day-old SPF chicks, with punctate hemorrhage in the larynx, numerous urate deposits in the ureter and kidney swelling. Meanwhile, there were no obvious gross lesions in chicks inoculated with the rYN-S2/RRKR strain, just as in the negative group (Figure 2B, right panels).

The clinical signs and necropsy results of pathogenicity experiments on SPF chicks of different ages revealed that the furin-S2′ site increased the capacity of IBV-rYN to cause neurological signs in SPF chicks of different numbers of days old, while the mortality and severity of neurological signs were age-dependent. However, the furin-S2′ site decreased the damage in the respiratory and urinary systems.

#### 3.2.3. Histopathological Examination

**Figure 3:**
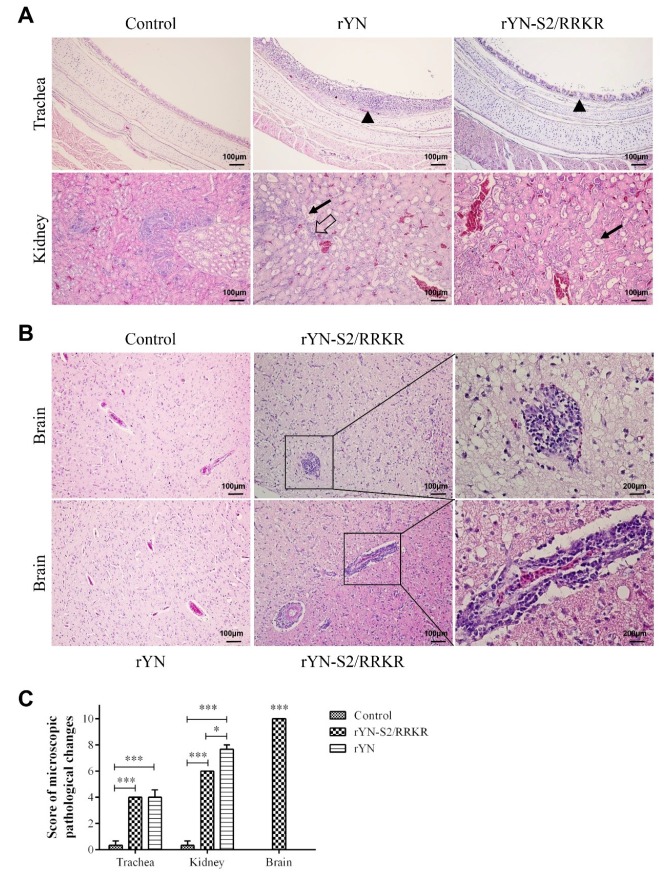
Microscopic lesions were observed in tissues of 1-day-old SPF chicks at 9 dpi. (**A**) Clear lesions could be observed in trachea and kidney in both virus-inoculated groups. The black triangles indicate clear mucosal thickening and infiltration of a large number of inflammatory cells in the trachea. The black arrows indicate cellular cast in the kidney. The blank arrow indicates inflammatory cell infiltration in kidney interstitium. (**B**) Results of H&E-stained brain sections from the rYN-S2/RRKR group. The panel indicates substantial microglial hyperplasia, forming microglial nodules in the brain (top). The panel indicates the formation of perivascular inflammatory infiltrates (bottom). No obvious lesions were observed in the brain of rYN and control groups (left). (**C**) Scores of 1-day-old chicken group. Sections stained with H&E were evaluated and the scores from 0 to 10, reflecting the severity of the lesions, were recorded. The indications for the scores were as follows: 0 = no microscopic lesions, 1–3 = mild lesions, 4–6 = moderate lesions and 7–10 = severe and extensive lesions. Both rYN and rYN-S2/RRKR caused serious injury in the respiratory and urinary systems of 1-day-old chicks, while rYN-S2/RRKR caused serious injury in the CNS (*n* = 4).

The microscopic lesions in samples of trachea and kidney were consistent with the gross lesions described above. In the rYN strain group, clear mucosal thickening was observed along with the infiltration of many inflammatory cells. Moreover, dilation of kidney tubules, inflammatory cell infiltration, hemorrhage in the kidney interstitium and cellular cast were detected in kidney of 1-day-old chicks (Figure 3A). In the rYN-S2/RRKR strain group, slight mucosal thickening and inflammatory cell infiltration were detected in the trachea of 1-day-old chicks. The dilation of kidney tubules and cellular cast could also be detected in kidney of 1-day-old chicks (Figure 3A). In brain samples, there were no lesions in brains from the rYN and negative control groups, while in the rYN-S2/RRKR group clear lesions were observed, such as substantial microglial hyperplasia, forming microglial nodules and perivascular inflammatory infiltrates (Figure 3B). We scored the severity of lesions according to the marking standard described in methods. The score results showed that both rYN and rYN-S2/RRKR could cause serious injury in the respiratory and urinary systems of 1-day-old chicks, but only rYN-S2/RRKR could significantly injure the CNS (Figure 3C).

**Figure 4:**
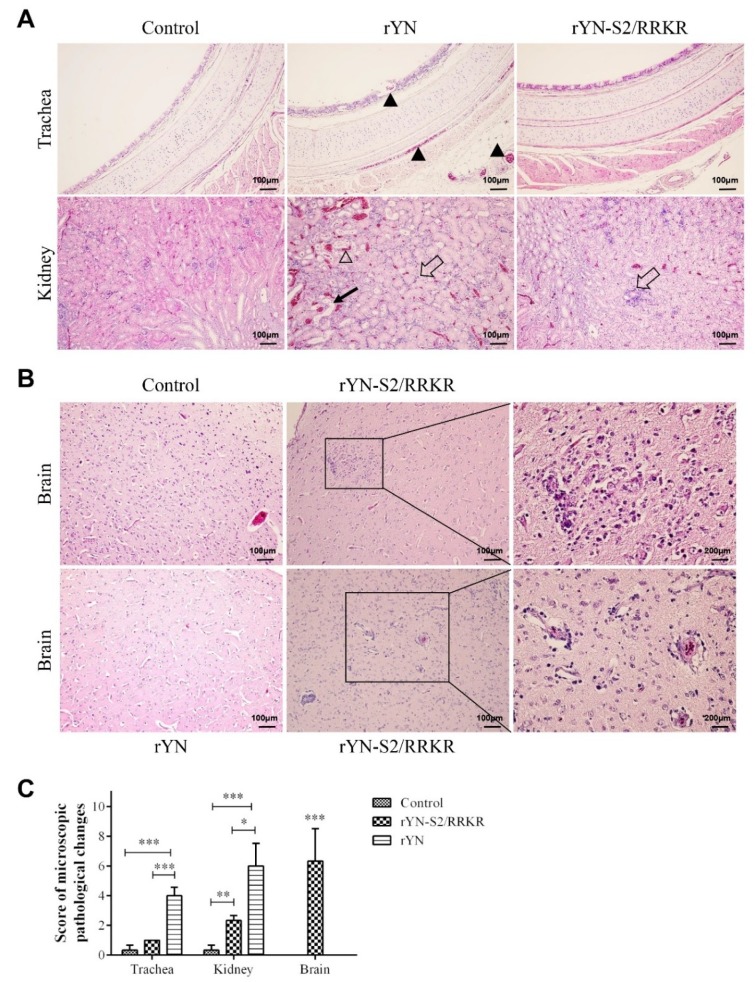
Microscopic lesions were observed in tissues of 3-week-old SPF chicks. (**A**) Clear lesions could be observed in the trachea and kidney in the rYN group. The black triangles indicate hemorrhage in mucosa and serosa in trachea. The blank triangle indicates hemorrhage in kidney interstitium. The black arrow indicates the cellular cast in kidney and the blank arrows indicate inflammatory cell infiltration in kidney interstitium. (**B**, Results of H&E-stained brain sections from the rYN-S2/RRKR group. The panel indicates substantial microglial hyperplasia in the brain (top). The panel indicates the formation of perivascular inflammatory infiltrates at a lower level than that in 1-day-old chickens (bottom). (**C**) Scores of the 3-week-old chicken group. Sections stained with H&E were evaluated and the scores from 0 to 10, reflecting the severity of the lesions, were recorded. The indications for the scores were as follows: 0 = no microscopic lesions, 1–3 = mild lesions, 4–6 = moderate lesions and 7–10 = severe and extensive lesions. rYN-S2/RRKR caused mild injury in the respiratory and urinary systems of 3-week-old chicks compared with rYN, but still caused severe injury in the CNS (n = 4).

In samples of 3-week-old chicks, hemorrhage in mucosa and serosa was seen in the trachea of the rYN-inoculated group, while there were no obvious lesions in the trachea of the rYN-S2/RRKR-inoculated and negative control groups. Dilation of kidney tubules, inflammatory cell infiltration, hemorrhage in the kidney interstitium and cellular cast were observed in kidney of the rYN-inoculated group. Conversely, only inflammatory cell infiltration was detected in kidney of the rYN-S2/RRKR-inoculated group (Figure 4A). There were no lesions in brains of the rYN and negative control groups, while substantial microglial hyperplasia and perivascular inflammatory infiltrates were detected in brains of the rYN-S2/RRKR-inoculated group (Figure 4B). The score results showed that rYN-S2/RRKR caused mild injury in the respiratory and urinary systems of 3-week-old chicks compared with rYN and severe injury in the CNS (Figure 4C).

The results of histopathological examination confirmed our previous speculation that rYN-S2/RRKR infection causes high mortality because of severe encephalitis. The formation of perivascular inflammatory infiltrates indicates that the blood–brain barrier (BBB) was broken and the central nervous system (CNS) was damaged.

#### 3.2.4. Immunohistochemistry Examination

**Figure 5:**
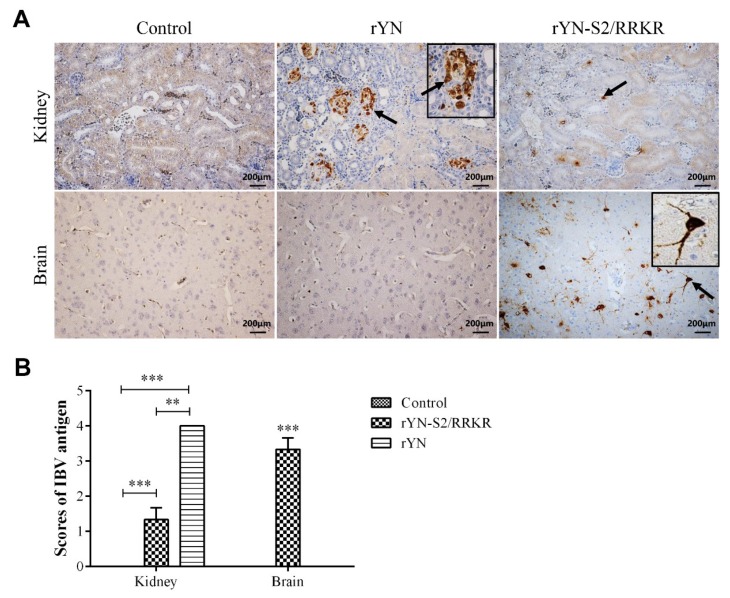
Results of IHC. (**A**) Results of IHC for IBV antigen in kidney and brain sections. The presence of rYN or rYN-S2/RRKR antigen was detected in the renal tubular epithelial cells, while substantial IBV antigen was detected in neurons of the brain from the rYN-S2/RRKR-inoculated group. (**B**) The detection of IBV was evaluated as the number of positive cells per section from each microscopic field through a ranked score of 0–4. The indications for the scores were as follows: 0 = no positive cells, 1 = 1–10 positive cells, 2 = 11–30 positive cells, 3 = 31–50 positive cells and 4 = > 50 positive cells. There were more rYN-positive cells in the kidney than for rYN-S2/RRKR, while there were no positive cells in brains of both rYN and negative control groups. There were obvious rYN-S2/RRKR-positive cells in brains (*n* = 3, right).

Because of the apparent change of tissue tropism, we determined the cell tropism by IHC. The results revealed the presence of rYN and rYN-S2/RRKR antigen in the renal tubular epithelial cells from rYN- or rYN-S2/RRKR-inoculated groups, while a large number of IBV antigen-positive cells were detected in the brain of the rYN-S2/RRKR-inoculated group. Additionally, the presence of rYN-S2/RRKR antigen was detected in neurons—not microglia or other glia (Figure 5A). We evaluated the number of positive cells as a marking standard as described above. The results showed that there were more rYN-positive cells in kidney than for rYN-S2/RRKR, while there were no positive cells in brains of both the rYN and negative control groups. There were significant numbers of rYN-S2/RRKR-positive cells in brains (Figure 5B). These observations showed that rYN-S2/RRKR is neurotropic and decreases epithelial tropism in vivo. In other words, the furin-S2′ site led the IBV-rYN strain to exhibit neurotropism, which has never previously been reported in IBV.

### 3.3. QX-type IBV Vaccine Can Still Provide Efficient Protection against rYN-S2/RRKR

Owing to the high pathogenicity of rYN-S2/RRKR to SPF chickens, we analyzed its antigenicity by neutralization assay and conducted an immunoprotection experiment. An original serum against rYN could neutralize both rYN and rYN-S2/RRKR efficiently in SPF chicken embryos and the 50% protective dose (PD_50_) was the same: 2^8.5^ PD_50_/0.1 mL. No chickens vaccinated with SZ130 showed any clinical signs after inoculation with rYN or rYN-S2/RRKR during the observation period (Table 2). Although there were clear differences in pathogenicity including clinical signs, tissue tropism and mortality between rYN and rYN-S2/RRKR, QX-type IBV vaccine can protect SPF chickens from rYN-S2/RRKR.

## 4. Discussion

The results of this study first demonstrated that a furin cleavage site immediately upstream of the fusion peptide in the S protein of QX-type IBV is related to the pathogenesis of IBV in brain. Virulent QX-type IBV with the furin-S2′ site caused severe encephalitis instead of nephritis or tracheitis, which has never been previously reported. Fortunately, although the furin-S2′ site increases the pathogenicity and changes the cell tropism of classical QX-type IBV, it does not change the antigenicity. The results of the neutralization test and immunoprotection experiment showed that original serum and vaccine can still provide effective protection in vivo and in vitro.

Although little research about the S2′ site has been performed, this site has been proven to play an important role in all virus stages, including attachment, virus–cell fusion and adaptation to its host cell. As for β-CoVs, the S2′ site is important for the invasion of susceptible cells [22,31]. For γ-CoVs, whose representative virus is IBV, the furin-S2′ site is the determinant of adaptation to Vero cells of the IBV-Beaudette strain. In the absence of this site, the IBV-Beaudette strain no longer grows in these cells. Our results confirmed that the furin-S2′ site plays an important role in the pathogenicity of IBV, besides adaptation to its host cell. The QX-type IBV possessing an S protein with a furin-S2′ site upstream of its FP can break the BBB and cause encephalitis.

**Figure 6:**
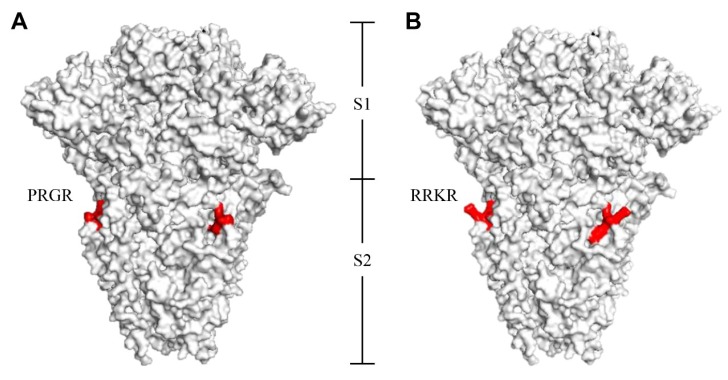
Structure of the S protein was predicted. We used homologous modeling to predict the structure of original and mutant S proteins according to the Cryo-EM structure of IBV S protein.

Some studies have indicated that MHV-JHM can infect oligodendrocytes, astrocytes and microglia, which are responsible for secreting cytokines and chemokines such as IFN-I and C-X-C motif chemokine 1 (CXCL1) following infection [6,32]. Additionally, neutrophils contribute to defense through the release of matrix metalloprotease 9 (MMP9), which helps increase the permeability of the BBB, resulting in an increase of specific T cells infiltrating into the CNS [33]. As for another neurotropic coronavirus, HCoV-OC43, astrogliosis and microgliosis are observed when it infects the brain [10]. A similar mutation was also observed in the S protein of HCoV-OC43, of which a furin-like cleavage site appeared at amino acid 758 between the S1 and S2 portions [34,35]. Conversely, this mutation leads to decreased neurovirulence and limited dissemination within the CNS. Our data from pathogenicity experiments clearly show that IBV with the furin-S2′ site can infect neurons and cause encephalitis, which has never previously been reported in IBV. Our results extend previous work on the function of the S protein of coronavirus. Based on this, we used homologous modeling to predict the structure of original and mutant S proteins according to the structure of IBV S protein (PDB accession number: 6CV0) (Figure 6) [36]. We found that the S2′ site is located on the surface of the S protein and can be modified by furin. We speculate that modification by furin may affect the fusion of virus with cells, which mildly damages the trachea and kidney; meanwhile, IBV with mutant S protein can infect neurons.

In summary, our results demonstrate that the furin cleavage site upstream of the FP in S protein is an important site for CoV, modulating entry, cell–virus fusion, adaptation to its host cell, cell tropism and pathogenicity, but not antigenicity. It remains unclear whether the neurotropism caused by the furin-S2′ site is IBV strain-dependent. Because of the mutability of the coronavirus genome, such as IBV-Beaudettte strain and mutant HCoV-OC43 with a furin-like site in the S protein, it is possible that mutant QX-type IBV would appear naturally and cause great economic losses in the poultry industry because of its high pathogenicity to chickens.

## Figures and Tables

**Table 1:** Time of death of embryonated eggs inoculated with rYN or rYN-S2/RRKR.

Passage	Virus	
	rYN	rYN-S2/RRKR
P1	144 h × 3 ^1^	36 h × 3
P2	144 h × 3	24 h × 3
P3	144 h × 3	24 h × 3
P4	120 h × 3	24 h × 3
P5	120 h × 2, 96 h × 1	24 h × 3

^1^ no. means the number of eggs died at the specific timepoint post inoculation within three eggs every passage.

**Table 2:** Experimental design and results of immunization and challenge test.

Group	No. of Chickens	Age (d)	Vaccine	Route and Dose of Inoculation	Challenge	Route and Dose of Challenge	Mortality (%)
A	10	1	SZ130	Intraocular, 10^5.0^EID_50_/0.2 mL	rYN	Intraocular, 10^5.0^EID_50_/0.1 mL	0/10 (0)
B	10	1	SZ130	Intraocular, 10^5.0^EID_50_/0.2 mL	rYN-S2/RRKR	Intraocular, 10^5.0^EID_50_/0.1 mL	0/10 (0)
C	10	1	SZ130	Intraocular, 10^5.0^EID_50_/0.2 mL	PBS	Intraocular, 0.1 mL	0/10 (0)
D	10	1	PBS	Intraocular, 0.2 mL	rYN	Intraocular, 10^5.0^EID_50_/0.1 mL	1/10 (10)
E	10	1	PBS	Intraocular, 0.2 mL	rYN-S2/RRKR	Intraocular, 10^5.0^EID_50_/0.1 mL	2/10 (20)
